# Radiotherapy for Symptomatic Knuckle Pads Associated With Dupuytren’s Disease: A Report of a Case Series

**DOI:** 10.7759/cureus.85796

**Published:** 2025-06-11

**Authors:** Amanda Stark, Yasamin Sharifzadeh, Jenna Kahn, Jessica Schuster, Elisabeth Weiss

**Affiliations:** 1 Radiation Oncology, Virginia Commonwealth University Health, Richmond, USA; 2 Radiation Oncology, Mayo Clinic, Rochester, USA; 3 Radiation Oncology, Kaiser Northwest (NW) Permanente, Portland, USA; 4 Radiation Oncology, University of Wisconsin-Madison, Madison, USA

**Keywords:** benign disease radiotherapy indication, dupuytren's disease, garrod's node, knuckle pad, palmar fibromatosis, radiation therapy

## Abstract

Purpose: Knuckle pads are often diagnosed in patients with palmar fibromatosis or Dupuytren’s disease. At present, no widely accepted treatment for knuckle pads has been established. Here, we describe the outcomes of patients with knuckle pads treated with radiotherapy at our institution.

Materials and methods: Patients who had received radiotherapy for knuckle pads since 2012 and completed questionnaires at the time of consultation and at follow-up interview are included in this longitudinal analysis. We report patient characteristics, treatment plans, patient-reported knuckle pad response to radiotherapy, and patient satisfaction.

Results: Nine Caucasian patients, median age 56 years old (range, 42-63), including seven women and two men, completed radiotherapy to their knuckle pads. Eight patients received simultaneous radiotherapy to the palms, and two patients additionally received radiotherapy to the soles. The time since treatment varied from 3 to 12 years, with a median duration of nine years. Typical treatment included a split-course prescription to 30 Gy in 10 daily fractions with a 6-12-week mid-treatment break. Electron beam energy was typically 6 MeV prescribed to the 90%-100% isodose line using a 1-1.5 cm bolus. Following radiotherapy, seven out of nine (78%) patients reported either resolution, flattening of knuckle pads, or stable disease. One patient (11%) reported resolution of knuckle pads in one hand, but enlargement and the development of new knuckle pads in the other hand. One patient (11%) reported worsening disease with enlargement of knuckle pads wrapping around the fingers toward the palmar aspect. Regarding symptomatic treatment response, eight patients (89%) reported improved symptoms or symptom resolution since radiotherapy including improvement in tenderness and pain, one (11%) reported stable symptoms. Minimal acute and chronic toxicity was reported. Eight patients (89%) reported favorable outcomes.

Conclusions: Our study details the treatment response of nine patients with knuckle pads to radiotherapy. Patients tolerated treatment well, with the majority reporting symptomatic and physical improvement, as well as favorable satisfaction with outcomes. Our findings suggest that radiotherapy is a safe and effective treatment option for knuckle pads providing durable disease control and can be considered in the appropriate clinical setting.

## Introduction

Knuckle pads, or Garrod’s nodes, are areas of benign subcutaneous fibromas overlying the extensor side of fingers and toes, most often involving the proximal interphalangeal joints. Knuckle pads have been associated with various conditions, including repeated trauma or friction to the knuckles, or can be idiopathic [1]. In adults, knuckle pads are often diagnosed in patients with palmar fibromatosis, also called Dupuytren’s disease or Dupuytren’s contracture [2-5]. Knuckle pads have, therefore, also been called dorsal Dupuytren’s nodes [6]. The reported incidence of knuckle pads in patients with palmar or plantar fibromatosis (Ledderhose disease) varies between 2.4% and nearly 50% [5-13].

Knuckle pads often cause no symptoms but can occasionally lead to discomfort or even pain. Large knuckle pads can also be cosmetically concerning, limit the range of finger motion, and cause restrictions to daily activities. The diagnosis of knuckle pads is typically made clinically. On clinical exam, the nodule is mobile over the joint and adheres to the skin [5] with no sign of synovitis. All fingers can be involved, predominantly the index finger [10], whereas the thumb is affected less often. Involvement of the metacarpophalangeal joint or the tissue between joints is less frequent.

At present, no widely accepted treatment for knuckle pads has been established. Various treatment options have been described mostly in the form of individual patient reports, often in children and adolescents. While knuckle pads might have been included in previous radiotherapy series reporting treatment for patients with palmar or plantar fibromatosis, no dedicated report on the outcome of radiotherapy for symptomatic knuckle pads is available to the best of our knowledge. Therefore, we report our institutional experience on radiotherapy for symptomatic knuckle pads in patients with palmar or plantar fibromatosis in this case series. Patient-reported outcomes of symptomatic response, change in knuckle pad size, and overall satisfaction with treatment outcome are described.

## Materials and methods

Patients diagnosed with palmar or plantar fibromatosis who had received radiotherapy for knuckle pads at our institution since 2012 and who had enrolled in an institutional review board (IRB)-approved protocol were contacted regarding outcomes. Patients’ demographic and clinical characteristics, as well as treatment parameters, were collected from clinical and radiotherapy charts. Patients had initially completed an IRB-approved questionnaire at the time of the consult. For this analysis, using the same questionnaire, patient-reported outcomes, including symptomatic and knuckle pad responses, as well as patient satisfaction, were recorded via phone interview or in person when patients returned for a new course of radiotherapy for newly developed disease manifestations outside the previous radiotherapy fields.

Nine Caucasian patients, median age 56 years (range, 42-63), including seven women and two men, completed radiation therapy to their knuckle pads. Their symptoms included tenderness, pain, or strain in all nine patients (100%), burning or itching sensation in two of nine (22%), inability to make a fist and interfering with activities of daily living such as cooking, gardening, and grasping items, and writing in three of nine (33%). No patient reported excessive physical stress on their hands, and four worked on computers for professional reasons. One patient had a diagnosis of type 1 diabetes and had a history of frozen shoulder. Another patient reported elbow tendinitis. Two patients were former cigarette smokers, and seven patients reported occasional alcohol use. Four patients had a family history of palmar fibromatosis, one patient was adopted, and therefore, the family history was unclear. One patient reported prior injury to a hand treated with surgery 2-10 years before radiotherapy for palmar fibromatosis of the ipsilateral hand and knuckle pads of the contralateral hand. Another patient had a steroid injection of the ipsilateral hand for trigger finger before radiotherapy. Patients were offered radiotherapy for symptomatic knuckle pads in the absence of established alternative treatment options while undergoing radiotherapy for early-stage Dupuytren's disease and Ledderhose disease (eight patients) or upon diagnosis with Dupuytren's disease (one patient).

## Results

Eight patients received simultaneous radiotherapy to the palms, and two additionally to the soles for palmar/plantar fibromatosis; one patient underwent radiotherapy to the palms at a later time. Follow-up since treatment for knuckle pads to analysis ranged from 3 to 12 years (median, nine years). Treatment was delivered on linear accelerators using a split-course prescription (6-12-week mid-treatment break) to 30 Gy in 10 daily fractions in eight patients and 27 Gy in nine fractions in one patient. The use of a mid-treatment break was based on the typical radiotherapy approach for early-stage Dupuytren's disease. The length of the treatment break varied based on patient preference and was modified over the years based on available literature [6,9]. Electron beam energy was 6 MeV except in one patient with thick knuckle pads wrapping around the fingers, where 12 MeV electron energy was used (Table 1).

**Table 1:** Treatments plans and individual patient outcome IDL: isodose line; PIP: proximal interphalangeal; RT: radiotherapy; R: right; L: left

Patient	Age (years)	Gender	PIP joint treated	RT details (electrons)	Simultaneous RT to palms or soles	Years since RT	Response	Patient satisfaction	Toxicity	Repeated RT
1	56	Female	R: 4th-5th; L: 3rd	R: 9 MeV, L: 12 MeV 1 cm bolus; 90% IDL	No	12	New knuckle pads, wrapping around fingers and spreading to the palmar surface	Made no difference	Tenderness acutely, now resolved	Yes: palms five years later
2	63	Female	L: 2nd-5th	6 MeV; 1 cm bolus; 95% IDL	Bilateral palms	12	Knuckle pads flattened, now asymptomatic	Very Satisfied	No	No
3	61	Female	L: 2nd	6 MeV; 1 cm bolus; 100% IDL	Bilateral palms	11	Knuckle pad resolved, no symptoms	Very satisfied, would highly recommend	No	No
4	53	Female	R: 5th	6 MeV; 1 cm bolus; 90% IDL	Bilateral palms	9	Knuckle pad and tenderness resolved	Very satisfied	Dry skin after treatment	No
5	42	Female	L: 2ndand 4th	6 MeV; 1 cm bolus; 90% IDL	Right palm	9	No further progression and no symptoms	Satisfied	No	Yes: outside field (left 5th digit, palm, left plantar foot)
6	44	Male	L: 4th	6 MeV; 1 cm bolus; 90% IDL	Bilateral palms	9	Knuckle pads are smaller, and symptoms have resolved	Super happy and very satisfied; would recommend to anyone considering	No	Yes: outside field (palms)
7	58	Female	L: 2nd	6 MeV; 1 cm bolus; 90% IDL	Bilateral palms and left foot	9	Stable knuckle pads, symptoms responded well, no evidence of progression	Very satisfied; would do it again	No	No
8	57	Female	R: 2nd-4th; L: 3rd-4th	6 MeV: 1.5 cm bolus; 90% IDL	Bilateral palms and soles	6	No overall change in appearance of the right hand, enlarging and new knuckle pads on the 2nd and 5th finger of the left hand, less painful on the right and left sides	Overall satisfied because it slowed progression	No	No
9	42	Male	L: 5th	6 MeV; 1.5 cm bolus; 100% IDL	Left palm	3	Knuckle pads resolved since radiotherapy, with no new pads	Best decision of my life. 250% satisfied	Knuckles are more sensitive	No

**Figure 1:**
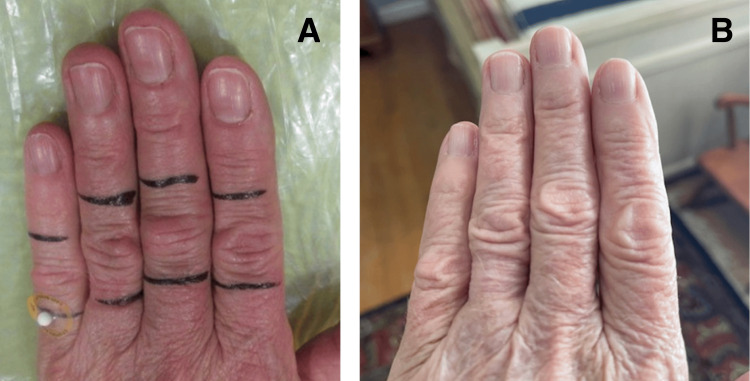
Knuckle pads before and after radiotherapy. Patient example with treatment field covering dorsal proximal interphalangeal joints (A) and 12 years later (B)

Depending on the thickness of the knuckle pads, the dose was prescribed to the 90%-100% isodose line using a 1-1.5 cm bolus. All patients underwent computed tomography to assess the lesion thickness and depth of the palmar fibromatosis as part of the treatment planning process. Monitor units were usually calculated manually per departmental electron depth dose curve tables. In patients where fingers were also treated from the palmar side for Dupuytren's disease involvement, composite plans were created in a commercial treatment planning system using dedicated electron calculation algorithms (Pinnacle, Philips Healthcare, The Netherlands; Eclipse, Varian, USA). To ensure an appropriate dose prescription and avoid overdosage through exit dose, the initial dose to the larger palmar field was calculated to achieve the prescribed dose. Then, the electron field covering the knuckle pads was added in the treatment planning system. To obtain adequate prescription dose coverage, i.e., total dose of 30 Gy, the dose calculation included the exit dose of each field. This composite planning process resulted in a reduction of the dose delivered through the knuckle pads field while maintaining the prescription dose and avoiding overdosage for both the palmar and knuckle pads target areas. Figure 1 shows a patient example before and after radiotherapy.

**Figure 2:**
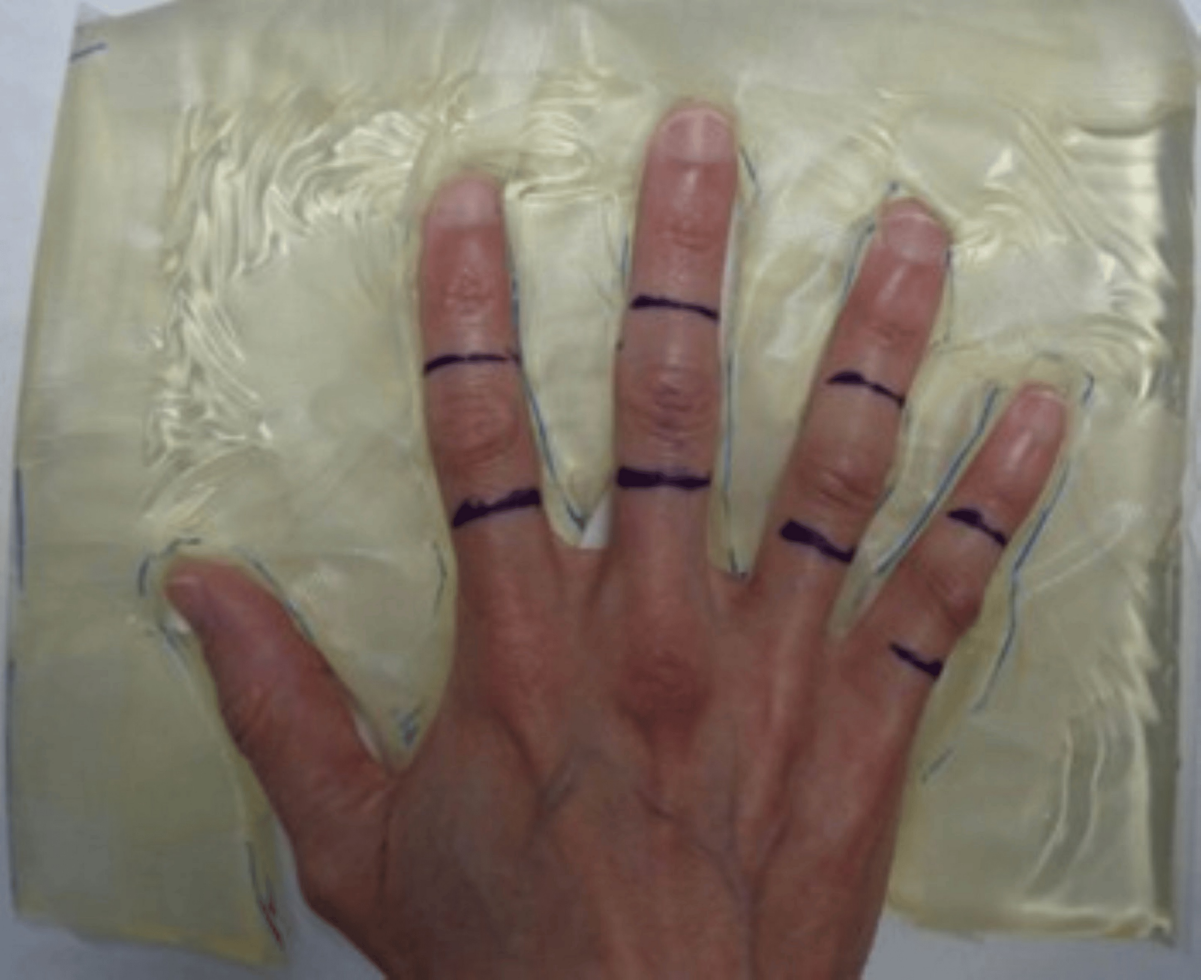
Patient setup Example of patient setup with individually cut out bolus material embedding the hand to separate fingers, thereby allowing sparing of uninvolved fingers, if needed. For treatment, a layer of bolus will be placed over the treatment area. Treatment fields encompass the knuckle pads with a 1-1.5 cm margin in proximal and distal directions while covering the whole width of the involved finger

Figure 2 displays the treatment setup for one patient. The patient is standing at the side of the treatment couch of the linear accelerator with the arm extended and the hand pronated. Reproducibility of daily alignment was checked visually based on skin markings, alignment with individual field blocks through the light field on the linear accelerator, and comparison with photo documentation of the initial treatment setup. The delivered dose is measured using in vivo dosimetry during the first fraction via an optically stimulated luminescent dosimeter.

**Figure 3:**
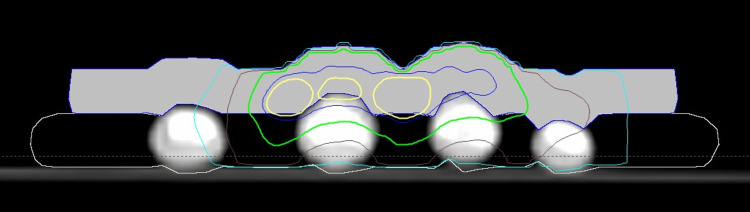
Example of electron dose distribution for knuckle pad treatment Using 6 MeV electrons prescribed to the 95% isodose line, knuckle pads of fingers two to five were treated using a 1 cm bolus between (beige outline) and a 1 cm bolus on top of the knuckle pads (solid light gray structure). The dose distribution shown here is at the level of the third finger knuckle pad (isodose lines: yellow, 95%; dark blue, 90%; green, 75%; brown, 50%; and light blue, 25%). Bolus outlines were manually created in the treatment planning system. The dose distribution for the second, fourth, and fifth finger knuckle pads is shown only partially, as knuckle pads in these fingers show in different CT planes CT: computed tomography

Figure 3 shows an example of the dose distribution created in the treatment planning system.

Seven of nine (78%) patients reported resolution (three patients, 33%), flattening and shrinkage of knuckle pads (two patients, 22%), or stable (no progression) disease (two patients, 22%). One patient (11%) reported resolution of knuckle pads in one hand, as well as enlargement and the development of new knuckle pads in the other hand. Another patient (11%) reported worsening disease with the enlargement of knuckle pads wrapping around the fingers toward the palmar aspect. Regarding symptomatic treatment response, eight patients (89%) reported improved symptoms or symptom resolution after radiotherapy, including improvement in tenderness and pain; one patient (11%) reported stable symptoms. Minimal acute and chronic toxicity was reported, including temporary skin dryness in one patient and increased tenderness in two patients, with resolution in one of them. Regarding patient satisfaction, eight (89%) patients reported favorable outcomes. Three patients had an additional course of radiotherapy for new manifestations of palmar or plantar fibromatosis, and one of them had additional treatment for knuckle pads in a previously uninvolved finger. Included in these three patients is the patient who experienced the progression of knuckle pads. This patient was treated at another clinic, and it is unknown whether knuckle pads were irradiated again at that time. None of the other patients received additional treatment for irradiated knuckle pads at a later time.

## Discussion

Knuckle pads are a common diagnosis in patients with palmar fibromatosis [5-12]. Knuckle pads have been described as a predisposing or risk factor for palmar and plantar fibromatosis [9,11]. Abe et al. [13] developed a risk score for recurrence and extension of Dupuytren’s disease, where the presence of knuckle pads, the presence of plantar fibromatosis, and radial side involvement in the palm showed a particularly high association with recurring Dupuytren’s disease.

Knuckle pads are typically diagnosed based on inspection and clinical symptoms, and further evaluation, including biopsy or ultrasound, is rarely performed but can be indicated to differentiate knuckle pads related to Dupuytren's disease from other conditions. Pathological exams found that dorsal knuckle pads and Dupuytren's disease are fibrosing disorders with common features [2,14], showing spindle-shaped myofibroblasts [15,16]. Ultrasound shows well-defined subcutaneous hypoechoic masses without internal flow signals at color Doppler and without bone or synovial involvement [16-18]. The differential diagnoses include calluses, gouty tophi, fibromas, foreign body reactions, and Heberden and Bouchard nodules, among others [18,19].

Numerous knuckle pad treatments have been attempted with varying success rates; none have proven to be a standard treatment approach. Resection has not been useful due to the risk of scarring and recurrence, whereas a case using erbium:yttrium-aluminum-garnet laser treatment was found to be successful [20-22]. Topical applications of urea lotion and salicylic acid were observed to be effective in individual cases [1,22]. A combination therapy of topical crisaborole, triamcinolone, and neomycin was reported to reduce knuckle pads in a patient who was previously treated with steroid injections and topical steroid treatment [23]. In another young patient, cantharidin‐podophyllotoxin‐salicylic acid application resulted in a response [24]. Other approaches that appeared to be successful in individual patients were carbon dioxide freezes combined with intralesional steroid injection; however, discomfort with this treatment was reported, and therefore, treatment was discontinued [25]. Intralesional 5-fluorouracil was reported to shrink knuckle pads in two patients [26], as shown in Table 2.

**Table 2:** Review of literature on treatment options for knuckle pads CPS: cantharidin-podophyllotoxin-salicylic acid; NA: not available; P: patient

Study	Number of patients (age/sex)	Follow-up	Treatment	Response	Toxicity	Previous treatment for knuckle pads
Sogliani et al. [1]	1 (15/male)	Years	Topical applications 25% urea-based cream bid, 40% urea-based cream overnight, and 30% salicylic acid ointment QD for 4-6 months	Resolved (fingers)/improved (toes)	None reported	Previous unsuccessful treatment with lower strength urea-based topicals, wart-removing products, and cryotherapy
Herold et al. [20]	1 (22/female)	18 months	Single treatment with erbium:yttrium-aluminum-garnet laser	Improved	None reported	Keratolytics are unsuccessful, recurrence after surgery
Paller and Hebert [22]	3 (P1: 4/male, P2: 14/female, P3: 9/female)	NA	P1: salicylic acid for three months; P2: 25% urea lotion for 1 months; P3: 0.025% fluocinolone acetonide over three months	P1: no change; P2: softening/flattening; P3: no change	None reported	P1: none reported; P2: cryotherapy with enlargement; P3: none reported
Liang et al. [23]	1 (45/male)	2 months	Topical 2% crisaborole ointment combined with triamcinolone acetonide and neomycin plaster bid for 2 weeks	Improved	None reported	Intralesional steroid injections several times and topical corticosteroids for three years unsuccessful
Hasbún et al. [24]	1 (15/male)	6 months	Topical 1% cantharidin, 5% podophyllotoxin, 30% salicylic acid (CPS) for 48 hours	Improved	Blisters	None reported
Allison and Allison [25]	1 (32/female)	7 years	Carbon dioxide freezes, combined with intralesional steroid injection	Some regression in size	Discomfort	None reported
Weiss et al. [26]	2 (27/female traumatic, 23/male occupational)	9 months/2 months	Intralesional fluorouracil 50 mg/mL (0.9 mL of fluorouracil with 0.1 mL of triamcinolone acetonide at 10 mg/mL)	Almost complete remission	None reported	None reported
Current patients	9 (42-63)	9 years	Radiotherapy	Eight improved or stable, one progression	Two increased tenderness, one dry skin	None

Radiotherapy is a well-established and effective treatment for early-stage palmar and plantar fibromatosis [11,27]. As demonstrated in our small patient series, knuckle pads often become symptomatic around the same time as Dupuytren’s disease; eight of our nine patients received simultaneous radiotherapy to the palms (and/or soles) and can be treated effectively with minimal toxicity during the same course using low-energy electron beams. Radiotherapy results in long-term symptom control in most patients.

While our report covers only a small heterogeneous patient cohort (more women than men) from our registry, the reported patients represent all patients treated for knuckle pads in our clinic during the reported time frame and, therefore, represent the actual real-world situation in our clinic. As many patients reside at a distance from our clinic, which prevents in-person follow-up visits, our results are primarily based on qualitative patient-reported outcomes. Prospective photographic documentation of change and employing objective measures of response during in-person visits would have provided a more refined outcome assessment. Nonetheless, patient satisfaction and patients' reporting of treatment results for this nonmalignant condition appear to be valuable endpoints, acknowledging that the retrospective nature of our study might introduce recall bias. Comparison of radiotherapy with other methods might also be helpful; however, as indicated, no alternative treatment options are established at this time. Further studies involving larger patient cohorts are suggested to support our findings. Such studies would also benefit from an assessment of the cost-effectiveness of this treatment method.

## Conclusions

Our study details the treatment response of nine patients with knuckle pads to radiotherapy. Patients tolerated treatment well, with the majority reporting symptomatic and physical improvement, as well as favorable satisfaction with outcomes. This is the report with the largest number of patients who received radiotherapy for their knuckle pads. It demonstrates the safety, tolerability, and efficacy of radiotherapy, providing durable disease control. Our findings suggest that radiotherapy is a safe and effective treatment option for symptomatic knuckle pads and may warrant consideration in the appropriate clinical setting.
